# Phenotypic associations of medical polypectomy and revision surgery following endoscopic sinus surgery: a retrospective study of a single-centre experience in Scotland

**DOI:** 10.1017/S0022215123000853

**Published:** 2023-11

**Authors:** Rasads Misirovs, Rory Chan, Kirsten Stewart, Brian Lipworth

**Affiliations:** 1 Tayside Rhinology Mega-Clinic and Scottish Centre for Respiratory Research, Ninewells Hospital and Medical School, University of Dundee, Scotland, UK; 2 Department of Doctoral Studies, Riga Stradins University, Riga, Latvia

**Keywords:** Corticosteroids, eosinophils, sinusitis, asthma, nasal polyps

## Abstract

**Background:**

Some chronic rhinosinusitis with nasal polyps patients undergo revision surgery at some point following initial functional endoscopic sinus surgery. This review aimed to identify the predictive factors for recurrence of nasal polyps requiring oral corticosteroids or revision surgery in chronic rhinosinusitis with nasal polyps following functional endoscopic sinus surgery.

**Method:**

A retrospective analysis of 221 patients who underwent functional endoscopic sinus surgery for chronic rhinosinusitis with nasal polyps in a tertiary rhinology centre, between January 2015 and December 2018, was undertaken.

**Results:**

Forty-four (21.6 per cent) patients underwent medical polypectomy, 19 (9 per cent) underwent revision surgery and 51 (24.3 per cent) underwent combined polypectomy during the mean follow-up time of 5.3 years. Patients aged less than 55 years of age, with a history of previous functional endoscopic sinus surgery, peripheral blood eosinophil counts of 300 cells/μl or higher, a Lund–Mackay score of more than 17 and concomitant aspirin-exacerbated respiratory disease had significantly increased odds for medical polypectomy, revision surgery and combined polypectomy.

**Conclusion:**

Knowing these predictive factors, clinicians can better identify patients with an increased likelihood of severe polyp recurrence and therefore arrange closer follow-up to optimise therapy.

## Introduction

Patients with chronic rhinosinusitis with nasal polyps are managed by general practitioners in the community, and by otorhinolaryngology surgeons, respiratory physicians and allergists in secondary care.^1^ The prevalence of chronic rhinosinusitis with nasal polyps is between 2 and 4 per cent.^2,3^ Current European Position Paper on Rhinosinusitis and Nasal Polyps 2020 (‘EPOS2020’) guidelines advocate a combined surgical and medical approach for the management of chronic rhinosinusitis with nasal polyps.^4^ Patients with chronic rhinosinusitis with nasal polyps most often complain of nasal obstruction and a reduced sense of smell, as well as anterior or posterior nasal discharge and a pressure sensation in the face.^1,5,6^

The management of chronic rhinosinusitis with nasal polyps is based on symptom management and a reduction of nasal polyp size. Current medical treatment involves topical corticosteroid sprays or drops, intranasal or systemic antihistamines in the presence of allergy, medical polypectomy with a short course of oral corticosteroids, and surgical polypectomy with functional endoscopic sinus surgery (FESS).^1,4^ The aim of FESS is to remove nasal polyps obstructing the nasal cavity, and to open the paranasal sinuses to improve drainage and access for intranasal corticosteroids.^1,5^ Recently, monoclonal antibodies such as dupilumab, mepolizumab and omalizumab have been approved for severe chronic rhinosinusitis with nasal polyps.^7^ Biologics are promising in terms of preventing the need for surgery and oral corticosteroids, but current costs are prohibitively high compared to standard medical and surgical treatment.^8,9^

Patients with severe chronic rhinosinusitis with nasal polyps may continue to experience troublesome symptoms with disease recurrence despite maximal medical therapy and FESS, requiring repeated courses of oral corticosteroids and/or revision FESS. Revision FESS indirectly reflects chronic rhinosinusitis progression and persistent type 2 inflammation.^10^

Clinicians and chronic rhinosinusitis with nasal polyps patients would benefit from having information available on specific risk factors that could help to predict the recurrence of nasal polyps requiring medical polypectomy or revision FESS, as currently there are no biomarkers that forecast who will respond to medical versus surgical treatment.^11^ The clinically most useful predictive factors for FESS effectiveness would be specific, sensitive, easy and usable.^10^ Allergic rhinitis, topical and systemic corticosteroid treatment, previous surgery for chronic rhinosinusitis, recurrent nasal polyps, and nasal polyps rich in eosinophils have all been associated with the need for recurrent chronic rhinosinusitis surgery.^10,12–14^ Hopkins *et al*. have reported higher Lund–Mackay scores (based on computed tomography (CT) sinus scans) as a predictive factor for revision FESS.^3^ Frequently, chronic rhinosinusitis with nasal polyps patients are separated into two groups depending on the presence or absence of asthma, as the latter has been reported to increase the need for revision FESS.^15,16^ Aspirin-exacerbated respiratory disease, or Samter's triad, has also been reported as a risk factor for revision FESS.^16^

Unlike medical care, past medical history and blood tests, the surgeon's expertise and skills are paramount but not easily assessed. Here, we looked at common phenotypes attributable to chronic rhinosinusitis with nasal polyps patients that are performed in standard rhinology clinical practice to see if these might help to predict the need for further medical polypectomy or revision surgery. By understanding these risk factors, patients can then be informed of the likely need for revision surgery or rescue oral corticosteroids in the post-operative period.^10,12^

## Materials and methods

### Patients

A retrospective review was undertaken of the medical case records of patients who underwent bilateral FESS between January 2015 and December 2018 (performed by three rhinologists) for surgical treatment of severe chronic rhinosinusitis with nasal polyps. Patients who underwent endoscopic polypectomy without their paranasal sinuses being operated on were not included in the study. Patients were identified via electronic operating theatre schedule records from the tertiary rhinology centre in Ninewells Hospital, Dundee, NHS Tayside, Scotland. Caldicott Guardian approval was granted for accessing and collecting the data (approval code: IGTCAL8446+).

**Figure 1.**
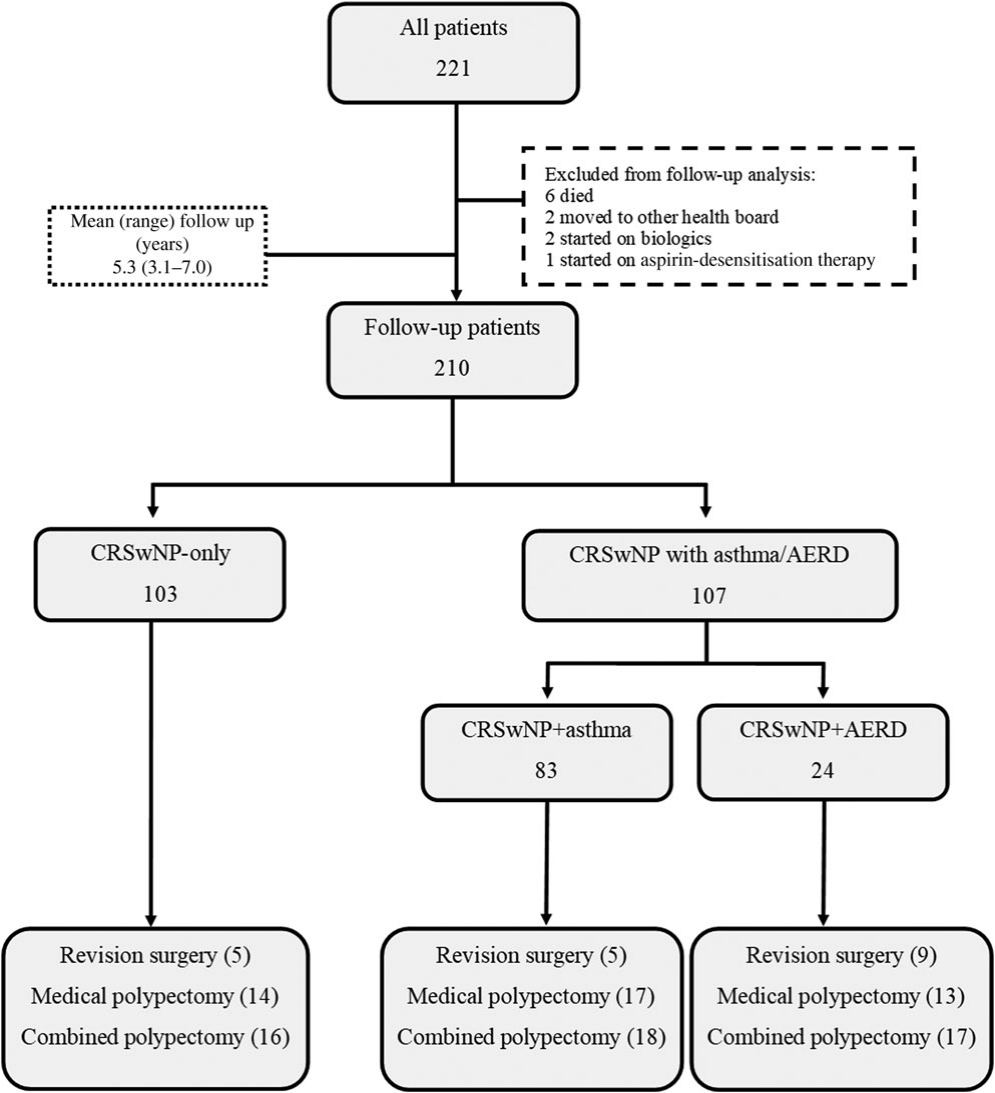
Patient flow chart. CRSwNP = chronic rhinosinusitis with nasal polyps; AERD = aspirin-exacerbated respiratory disease

Demographic, clinical history and examination data were available for 221 patients, allowing the follow-up of 210 patients (Fig. 1).

Diagnosis of chronic rhinosinusitis with nasal polyps was based on European Position Paper on Rhinosinusitis and Nasal Polyps 2020 criteria for symptoms, endoscopic and sinus CT findings. The exclusion criteria were patients taking regular systemic corticosteroids and medications that might affect chronic rhinosinusitis with nasal polyps, such as immunosuppressive drugs and monoclonal antibodies for conditions like cystic fibrosis, systemic vasculitis, rheumatoid conditions, asthma and others, as well as those undertaking aspirin desensitisation therapy for aspirin-exacerbated respiratory disease.

We divided our chronic rhinosinusitis with nasal polyps patients into three groups based on commonly presenting phenotypes in our rhinology clinic: chronic rhinosinusitis with nasal polyps with no asthma or aspirin-exacerbated respiratory disease (chronic rhinosinusitis with nasal polyps only), chronic rhinosinusitis with nasal polyps plus asthma, and chronic rhinosinusitis with nasal polyps plus aspirin-exacerbated respiratory disease. Patients were considered to have asthma if they had a physician diagnosis corresponding with Global Initiative for Asthma criteria. Patients with aspirin-exacerbated respiratory disease had a clinical triad of: chronic rhinosinusitis with nasal polyps, asthma, and a documented history of developing respiratory symptoms, angio-oedema or anaphylaxis following the ingestion of aspirin and/or non-steroidal anti-inflammatory drugs.

Nasal polyp scores were obtained from operation notes or from clinic letters when patients were consented for FESS. Modified Lildholdt grading was used, with the grading in each nasal passage being scored from 0 to 4 (0, no nasal polyps; 1, small polyps confined to the middle meatus; 2, moderate-sized polyps not crossing the lower edge of the inferior turbinate; 3, large polyps crossing the lower edge of the inferior turbinate; and 4, large polyps touching the floor of nasal passage), with a total maximum score of 8.

Severity of sinus inflammation was quantified by assessing the degree of sinus mucosal thickening on CT sinus imaging using the Lund–Mackay scoring system.^4^ Only CT sinus scans performed within a one-year period prior to FESS were included in the data analysis.

The average number of peripheral blood eosinophils was obtained from available blood test results at the time of diagnosis of chronic rhinosinusitis with nasal polyps and up to three years prior to FESS. Peripheral blood eosinophil results during acute hospital admissions were excluded because of the potential confounding effect of deteriorated health warranting admission and medical treatment (such as systemic or oral corticosteroids). Peripheral blood eosinophil counts taken within a three-month period following oral corticosteroid treatment were excluded. Total and specific immunoglobulin E (IgE) in response to common aeroallergens (house dust mite, cat, dog, grass, birch) were obtained from blood tests conducted any time before (since diagnosis with chronic rhinosinusitis with nasal polyps) or after FESS. The results of peripheral blood eosinophil counts, and total and specific IgE were taken from blood test reports requested by ENT doctors, as well as general practitioners, or respiratory or other doctors in the out-patient setting.

Details on the extent of FESS were obtained from operation notes.

Information on prescribed immediate post-operative intranasal corticosteroids was obtained from post-operative hospital discharge notes. Long-term intranasal corticosteroids history was obtained from post-operative ENT clinic notes.

Medical polypectomy was defined as receiving a minimum course, consisting of 5 days of at least 20 mg daily of oral prednisolone, that was not immediately planned as part of post-operative management, and which was indicated for chronic rhinosinusitis with nasal polyps, not for asthma in chronic rhinosinusitis with nasal polyps plus asthma patients or aspirin-exacerbated respiratory disease patients. Revision surgery was defined as either a revision FESS or simple endoscopic polypectomy during the follow-up period after the FESS. A course of oral corticosteroids as per pre-operative planning for revision surgery was not included as a separate medical polypectomy. Combined polypectomy was defined as having either medical polypectomy or revision surgery. Once a patient had received medical polypectomy, data were still collected on revision surgery, but once a patient had undergone revision surgery, no data were collected on medical polypectomy because having to undergo a revision surgery was the main study endpoint. For some patients, medical polypectomy was the alternative to revision surgery, especially during the global pandemic of coronavirus disease 2019; therefore, we decided to create this combined polypectomy endpoint.

Follow-up time was defined as the time from the initial FESS until 31 December 2021. Patients were actively followed up in the ENT clinic for up to three years following the FESS; if patients and ENT doctors were satisfied with the outcome, the patients were discharged. Following that, if they had not been referred back to our ENT department and still resided in the health board area that our ENT department covers, we assumed their chronic rhinosinusitis with nasal polyps was stable and did not require medical polypectomy or ENT review for consideration of revision surgery.

### Statistical analysis

Data analysis was performed using IBM SPSS® Statistics software version 27.0. Continuous variables were assessed for distribution of normality with normality plots. Data for total IgE were logarithmically transformed to achieve normal distribution, with results expressed as geometric means. Student's *t*-test was used to compare means between groups for continuous variables with normally distributed data. Fisher's exact two-sided test was used to compare groups for categorical variables. The Mann–Whitney test was used for nasal polyp score analysis as data were not normally distributed. A linear regression test was used to analyse the odds ratios for further medical polypectomy, revision surgery and combined polypectomy, with odds ratios adjusted for age and gender. Statistical significance was determined with a two-tailed alpha error of 0.05. Bonferroni corrections were applied when multiple tests were performed comparing the chronic rhinosinusitis with nasal polyps only group, the chronic rhinosinusitis with nasal polyps plus asthma group, and the chronic rhinosinusitis with nasal polyps plus aspirin-exacerbated respiratory disease group.

## Results

A total of 221 patients underwent various extents of FESS during the four-year study period (Table 1, in the supplementary material, available on *The Journal of Laryngology & Otology* website). Follow-up data on further medical polypectomy, revision surgery and combined polypectomy were available for 210 patients (Fig. 1). Overall, 44 (21.6 per cent) patients underwent further medical polypectomy, 19 (9 per cent) patients underwent revision surgery, and 51 (24.3 per cent) patients underwent combined medical and surgical polypectomy.

On discharge from hospital post-operatively, 198 (89.6 per cent) and 19 (8.6 per cent) patients were prescribed intranasal corticosteroids drops or spray, respectively. Following six-month post-operative review, 80 (36.2 per cent) patients remained on intranasal corticosteroids drops and 118 (53.4 per cent) patients continued taking intranasal corticosteroid spray (missing data for 23 (10.4 per cent) patients). Eighty-one patients (36.7 per cent) were taking antihistamines; of these, 78 patients (35.3 per cent) were taking oral antihistamines and 10 patients (4.5 per cent) were taking intranasal antihistamines. The mean (range) follow-up duration was 5.3 (3.1–7.0) years.

### Odds ratios for predicting medical and/or surgical polypectomy

**Figure 2.**
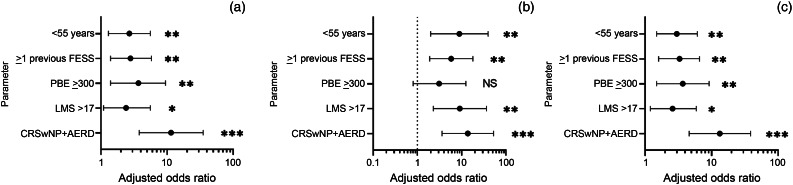
Adjusted odds ratio for (a) medical polypectomy, (b) revision functional endoscopic sinus surgery (FESS) and (c) combined polypectomy (odds ratios adjusted to age and gender). *p < 0.05; **p < 0.01; ***p < 0.001. PBE = peripheral blood eosinophils; LMS = Lund–Mackay score; CRSwNP = chronic rhinosinusitis with nasal polyps; AERD = aspirin-exacerbated respiratory disease; NS = non-significant (*p* ≥ 0.05)

Adjusted odds ratios for recurrence are shown in Fig. 2 and in Table 2 in the supplementary material (available on *The Journal of Laryngology & Otology* website). All phenotypic associations were significant predictors for medical and combined polypectomy aside from revision surgery alone with average peripheral blood eosinophil counts of 300 cells/μl or higher.

### Demographics

The mean patient age was 52.9 years (standard deviation = 13.8) and 74 per cent of the patients were male. There was no significant difference of gender proportion between the three groups based on having chronic rhinosinusitis with nasal polyps only, or chronic rhinosinusitis with nasal polyps plus asthma, or chronic rhinosinusitis with nasal polyps plus aspirin-exacerbated respiratory disease. More detailed demographic, clinical and follow-up differences between the chronic rhinosinusitis with nasal polyps groups without and with asthma (chronic rhinosinusitis with nasal polyps plus asthma, and chronic rhinosinusitis with nasal polyps plus aspirin-exacerbated respiratory disease) are summarised in Table 3 in the supplementary material (available on *The Journal of Laryngology & Otology* website). Furthermore, we show significant differences between chronic rhinosinusitis with nasal polyps plus asthma and chronic rhinosinusitis with nasal polyps plus aspirin-exacerbated respiratory disease in Table 4 in the supplementary material (available on *The Journal of Laryngology & Otology* website).

### Peripheral blood eosinophils

Peripheral blood eosinophil count values were available in 176 patients. There was no significant difference in average peripheral blood eosinophil counts between genders (*p* = 0.285). The mean (95 per cent confidence interval (CI)) of the average peripheral blood eosinophil count was 380 (348–412) cells/μl. There were significant differences in peripheral blood eosinophil counts between chronic rhinosinusitis with nasal polyps only and chronic rhinosinusitis with nasal polyps plus asthma and chronic rhinosinusitis with nasal polyps plus aspirin-exacerbated respiratory disease patients (Tables 3 and 4 in the supplementary material). A significant difference was observed for average peripheral blood eosinophil count values between chronic rhinosinusitis with nasal polyps only and chronic rhinosinusitis with nasal polyps plus aspirin-exacerbated respiratory disease patients (*p* < 0.001).

### Total immunoglobulin E

Total IgE values were available in 89 patients. The geometric mean (95 per cent CI) total IgE was 88 (66–116) kIU/l. There was a significant difference in geometric mean total IgE between males and females (106 and 55 respectively, *p* = 0.035). No significant difference was seen for geometric mean total IgE when comparing patients with a peripheral blood eosinophil count of 300 cells/μl or higher versus less than 300 cells/μl (96 and 79 kIU/l respectively, *p* = 0.512). A significant difference in geometric mean total IgE was found between chronic rhinosinusitis with nasal polyps only and combined chronic rhinosinusitis with nasal polyps plus asthma and chronic rhinosinusitis with nasal polyps plus aspirin-exacerbated respiratory disease patients (Table 3 in the supplementary material).

### Specific immunoglobulin E

Ninety-one patients had specific IgE tests carried out for common aeroallergens, and 52.7 per cent had a positive result (≥0.35 kU/l) to one or more allergen. No significant association was present with other variables.

### Nasal polyp score

Nasal polyp scores were available for 198 patients. The median (interquartile range) nasal polyp score was 6 (2). Men had higher median nasal polyp scores (interquartile range) than women (score of 7 (2) *vs* 6 (2) respectively, *p* = 0.004).

### Lund–Mackay score

Computed tomography sinus scans performed within one year prior to FESS were available in 178 patients. The mean (95 per cent CI) Lund–Mackay score was 13.3 (12.6–14.1). No significant difference in Lund–Mackay score was detected between genders (*p* = 0.878). Based on peripheral blood eosinophil count, significant differences in Lund–Mackay score were found between patients with average peripheral blood eosinophil count of 300 cells/μl or higher versus less than 300 cells/μl (scores of 14.2 and 11.7 respectively, *p* = 0.007). There were significant differences in Lund–Mackay score between chronic rhinosinusitis with nasal polyps only, chronic rhinosinusitis with nasal polyps plus asthma and chronic rhinosinusitis with nasal polyps plus aspirin-exacerbated respiratory disease patients (Tables 3 and 4 in the supplementary material).

### History of previous functional endoscopic sinus surgery

Overall, 132 (59.7 per cent) patients had not had previous FESS. There was a significant difference in average peripheral blood eosinophil count between patients who had previously undergone FESS and those who had not (424 *vs* 339 cells/μl respectively, *p* = 0.005). A non-significant difference in Lund–Mackay score was seen between these two groups (14.4 and 12.8 respectively, *p* = 0.053).

### Medical polypectomy

Twenty-eight men (22.3 per cent of all men) and 16 women (42.1 per cent of all women) had medical polypectomy, although the difference between the genders was not significant (*p* = 0.122). No differences in receiving medical polypectomy were shown between chronic rhinosinusitis with nasal polyps only and chronic rhinosinusitis with nasal polyps plus asthma patients (*p* = 0.207). A significant difference between chronic rhinosinusitis with nasal polyps only and combined chronic rhinosinusitis with nasal polyps plus asthma and chronic rhinosinusitis with nasal polyps plus aspirin-exacerbated respiratory disease group patients (Table 3 in the supplementary material) was present because of higher numbers of patients requiring medical polypectomy among the chronic rhinosinusitis with nasal polyps plus aspirin-exacerbated respiratory disease patients (Table 4 in the supplementary material).

There was a significant difference in the proportion of patients who received medical polypectomy when comparing patients aged less than 55 years versus those aged 55 years or older (28.7 and 13.5 per cent respectively, *p* = 0.01).

A significant difference in average peripheral blood eosinophil count was found between those patients who received medical polypectomy and those who did not (452 *vs* 342 cells/μl respectively, *p* = 0.002). There was a significant difference in the proportion of patients who received medical polypectomy based on their average peripheral blood eosinophil count of 300 cells/μl or higher versus a count of less than 300 cells/μl (28.7 and 11.5 per cent respectively, *p* = 0.011).

There was no significant difference in total IgE between patients who received medical polypectomy and those who did not (*p* = 0.385), as well as no significant difference in positive specific IgE to common aeroallergens between these two groups (*p* = 0.821).

### Revision surgery

Thirteen men (9.1 per cent) and six women (12.2 per cent) had further revision surgery. There were no significant differences between the genders (*p* = 0.589). Four of the patients underwent two revision surgical procedures during the follow-up period.

There were significant differences in the proportion of patients who underwent revision surgery between the chronic rhinosinusitis with nasal polyps only and chronic rhinosinusitis with nasal polyps plus aspirin-exacerbated respiratory disease patients (4.9 and 37.5 per cent respectively, *p* < 0.001), and between patients aged less than 55 years and those aged 55 years or more (15 and 2.1 per cent respectively, *p* = 0.001).

When comparing patients who underwent revision surgery with those who did not, there was no significant difference in average peripheral blood eosinophil count (*p* = 0.114). No significant difference was present in the proportion of patients who underwent revision surgery based on their average peripheral blood eosinophil count being 300 cells/μl or higher or less than 300 cells/μl (*p* = 0.261).

There was no significant difference in total IgE and positive specific IgE responses to common aeroallergens between patients who had and who did not have revision surgery (*p* = 0.753 and *p* = 0.526, respectively).

### Combined polypectomy

In total, 51 patients (24.3 per cent) underwent combined polypectomy (medical polypectomy and/or revision surgery). There were 33 men (21.3 per cent) and 18 women (32.7 per cent), with no significant differences between genders (*p* = 0.101). During the follow-up period, eight patients initially had medical polypectomy followed by revision surgery sometime later.

There were significant differences in the proportion of patients who had combined polypectomy when comparing chronic rhinosinusitis with nasal polyps only versus chronic rhinosinusitis with nasal polyps plus aspirin-exacerbated respiratory disease patients (15.5 *vs* 70.8 per cent respectively, *p* < 0.001), and when comparing patients aged less than 55 years versus those aged 55 years or older (32.7 *vs* 14.4 per cent respectively, *p* = 0.002).

When comparing patients who underwent combined polypectomy versus those who did not, there was a significant difference in average peripheral blood eosinophil count (459 *vs* 342 cells/μl respectively, *p* < 0.001), as well as a significant difference in the proportion of patients who underwent combined polypectomy based on average peripheral blood eosinophil counts of 300 cells/μl or higher or less than 300 cells/μl (31.4 *vs* 13.1 per cent respectively, *p* = 0.009).

No significant association was detected between total IgE and further combined polypectomy (*p* = 0.369).

## Discussion

The present study shows an increased likelihood of the need for medical polypectomy or revision surgery in younger patients with a history of previous FESS, higher peripheral blood eosinophil counts, a higher Lund–Mackay score and the presence of aspirin-exacerbated respiratory disease. For example, the odds ratios in patients with an average peripheral blood eosinophil count of 300 cells/μl or higher, indicate a 73 per cent significantly increased likelihood of requiring medical polypectomy and 73 per cent likelihood for combined polypectomy. Radiological Lund–Mackay scores of more than 17 were associated with an increased likelihood of medical polypectomy (58 per cent), revision surgery (89 per cent) and combined polypectomy (62 per cent).

We also observed significant differences in peripheral blood eosinophil count, total IgE values, nasal polyp scores and Lund–Mackay scores between chronic rhinosinusitis with nasal polyps patients depending on the presence or absence of asthma or aspirin-exacerbated respiratory disease. These markers of chronic rhinosinusitis with nasal polyps increased in severity, from being the lowest in chronic rhinosinusitis with nasal polyps only patients to the highest in chronic rhinosinusitis with nasal polyps plus aspirin-exacerbated respiratory disease patients.

There is conflicting evidence regarding an association of patient age with revision surgery during follow-up.^10^ We hypothesise that patients younger than 55 years of age have higher odds for revision surgery as they may be more fit for general anaesthetic compared to patients aged over 55 years. Following surgical and anaesthetic assessments, older patients with concerns about undergoing an operation requiring general anaesthetic may have opted against having surgery. Nevertheless, as we did not evaluate co-morbidities in each patient, this is merely speculative.

The mean age of patients in our cohort was 52.9 years. The incidence of nasal polyps increases with age, with a peak in the sixth decade; it is more common in men and less common before the third decade of life.^5,11,17^ In our cohort, only 4.5 per cent of patients were younger than 30 years of age, while there were more males than females. There might be other factors that explain why men were being operated on three times more. Physician bias has been reported as having an effect on decision-making in situations where the indications for surgery are less clear.^17^

Up to 50 per cent of patients with chronic rhinosinusitis with nasal polyps have co-existent asthma, which has been shown to be associated with more severe sinonasal disease.^4,11,15^ In our cohort, 51.1 per cent of patients had asthma or aspirin-exacerbated respiratory disease, 38.9 per cent had asthma, and 12.2 per cent had aspirin-exacerbated respiratory disease; these rates are similar to previous reports.^10,11,16^ Although not statistically significant, a higher proportion of female chronic rhinosinusitis with nasal polyps patients had aspirin-exacerbated respiratory disease compared to males, which has been reported before.^5,16,18^

The finding of higher Lund–Mackay scores when comparing chronic rhinosinusitis with nasal polyps only, chronic rhinosinusitis with nasal polyps plus asthma and chronic rhinosinusitis with nasal polyps plus aspirin-exacerbated respiratory disease patients supports the notion of more severe sinonasal inflammation. We should emphasise that chronic rhinosinusitis with nasal polyps plus asthma and chronic rhinosinusitis with nasal polyps plus aspirin-exacerbated respiratory disease are two distinct groups. As there are no *in vitro* tests available to diagnose aspirin-exacerbated respiratory disease, patients should be specifically asked whether nasal or respiratory symptoms are exacerbated by the intake of salicylates, non-steroidal anti-inflammatory agents, and dietary sources such as fresh berries and nuts.^5,11,16^

Chronic rhinosinusitis with nasal polyps plus aspirin-exacerbated respiratory disease patients were more likely to have had previous FESS, in keeping with previous literature.^11,12,16^ We suggest that higher levels of peripheral blood eosinophils might alert clinicians to the presence of possible aspirin-exacerbated respiratory disease, as chronic rhinosinusitis with nasal polyps plus aspirin-exacerbated respiratory disease patients had a higher peripheral blood eosinophil count. The concept of the unified airway suggests that upper airway inflammation may influence lower airway inflammation and vice versa.^12,15^ Medical and surgical treatment of chronic rhinosinusitis in patients with asthma has been shown to decrease asthmatic and sinonasal symptoms.^15^

Nineteen patients (9 per cent) in our cohort underwent revision surgery; more specifically, 4.9, 6 and 37.5 per cent underwent revision surgery for chronic rhinosinusitis with nasal polyps only, chronic rhinosinusitis with nasal polyps plus asthma and chronic rhinosinusitis with nasal polyps plus aspirin-exacerbated respiratory disease, respectively. Unsurprisingly, a history of previous FESS is associated with more severe sinus disease and has been reported as a factor associated with revision surgery, although Tosun *et al*. reported that previous FESS has no effect on post-operative recurrences.^12^ Somewhat similar results to our findings have been reported in the literature in aspirin-exacerbated respiratory disease patients, but the incidence of revision surgery in chronic rhinosinusitis with nasal polyps only and chronic rhinosinusitis with nasal polyps plus asthma patients differs.^10,12^ In our cohort, patients who underwent previous FESS had a higher peripheral blood eosinophil count.

Computed tomography scans are usually performed as part of surgical planning, but are also used for the assessment of chronic rhinosinusitis severity.^5^ No difference in Lund–Mackay score was present between genders in our cohort, which differs from other reports.^11^ Patients with a Lund–Mackay score of more than 17 had higher odds for requiring revision surgery, which corresponds with other reports of patients with higher CT scores and the failure of FESS at two years.^3^

Patients with aspirin-exacerbated respiratory disease were more likely to undergo revision surgery. Peripheral blood eosinophil count and total IgE did not differ among patients who did or did not undergo revision surgery.

There were fewer patients who received medical polypectomy in the chronic rhinosinusitis with nasal polyps only group compared to the rest of the patients who also had asthma and aspirin-exacerbated respiratory disease (Table 3 in the supplementary material). When analysed in more detail, however, the findings revealed no significant difference between the chronic rhinosinusitis with nasal polyps only patients and the chronic rhinosinusitis with nasal polyps plus asthma patients, but a significant difference between the chronic rhinosinusitis with nasal polyps plus asthma patients and the aspirin-exacerbated respiratory disease patients. We can therefore say that chronic rhinosinusitis with nasal polyps patients with asthma do not have higher risk for further medical polypectomy compared to chronic rhinosinusitis with nasal polyps only patients; it is aspirin-exacerbated respiratory disease that increases the risk for medical polypectomy.

More patients younger than 55 years of age received medical polypectomy compared to those older than 55 years. This could be partially explained by older patients having higher risk factors for potential side effects of oral corticosteroids, such as diabetes, osteoporosis and glaucoma, and this might have been the reason why they were prescribed medical polypectomy less frequently.

Patients with a higher peripheral blood eosinophil count were more likely to receive medical polypectomy, reflecting a higher type 2 inflammatory burden. This might have been influenced by surgeons being more likely to prescribe medical polypectomy to patients with a higher peripheral blood eosinophil count, as it is known that corticosteroids have better therapeutic effect in patients with blood eosinophilia.^4,12^ This is somewhat confirmed, as peripheral blood eosinophil count had no association with patients undergoing revision surgery.

Type 2 inflammation has been characterised by increased levels of the cytokines interleukin (IL)-4, IL-5 and IL-13 resulting in peripheral blood eosinophilia, elevated fractional exhaled breath nitric oxide and total IgE.^4,15^ Currently, tests of these cytokines are not commonly performed in rhinology or asthma clinics, but we believe peripheral blood eosinophil count and total IgE should be determined as part of standard clinical practice in patients with chronic rhinosinusitis with nasal polyps, as the results may be useful in guiding treatment and predicting prognosis.^5^ The question arises whether fractional exhaled breath nitric oxide, a specific marker of IL-13 expression, should be performed routinely in chronic rhinosinusitis with nasal polyps patients without known asthma in order to help stratify the type 2 inflammatory burden, although in our hospital it would not be feasible because of the current workload.^19,20^

We have chosen medical polypectomy and revision surgery as pragmatic endpoints, and assessed which patients ended up receiving them. We elected to combine medical polypectomy and revision surgery as a composite endpoint. In our rhinology clinic, medical polypectomy consists of a two-week course of oral prednisolone 25 mg tablet once a day with or without antibiotics. A Cochrane review of short-course oral corticosteroids for chronic rhinosinusitis has shown improvement in quality of life, less severe nasal symptoms and smaller nasal polyps that lasted up to three to six months, which is similar to a randomised clinical trial by Vaidyanathan *et al*. using a two-week course of 25 mg of prednisolone.^1,21^ Short-term therapy with oral corticosteroids should not be given more than twice per year because of the cumulative systemic burden.^6,22^ The most common short-term side effects of oral corticosteroids are gastrointestinal disturbances and insomnia, and the long-term side effects are osteoporosis, type II diabetes, weight gain, skin thinning, proximal myopathy, hypertension, cataracts, glaucoma and psychiatric disturbance.^23^

About 10–20 per cent of patients undergo a revision surgical procedure within five years.^3,10^ As per our results, patients with aspirin-exacerbated respiratory disease have higher odds for undergoing revision surgery; therefore, we suggest having a high level of suspicion for unreported aspirin-exacerbated respiratory disease in chronic rhinosinusitis with nasal polyps patients who require revision surgery, especially in patients with high eosinophil counts and a high Lund–Mackay score.^12^ Some studies report that having asthma or aspirin-exacerbated respiratory disease is not associated with revision surgery during follow-up.^10^ In our cohort, chronic rhinosinusitis with nasal polyps plus asthma patients did not have higher revision surgery rates, which is different from other reports.^11^ This is possibly because, in this study, chronic rhinosinusitis with nasal polyps plus asthma patients were carefully separated from nasal polyps plus aspirin-exacerbated respiratory disease patients. When patients have a diagnosis of aspirin-exacerbated respiratory disease, additional effort should be made to educate patients about non-surgical management options, perhaps including anti-leukotriene medication, aspirin desensitisation and/or dupilumab, and the overall long-term post-operative prognosis.^5,7,16^ Additionally, surgeons should consider performing more extensive FESS in patients at high risk for recurrence of nasal polyposis, as Hopkins *et al*. reported a significantly lower requirement for revision surgery when additional sinus surgery was performed during surgical polypectomy.^3^

Studies suggest that patients with prolonged and chronic disease might benefit more from surgery than continued medical therapy.^10^ This might be overturned by the availability of biologics based on phase 3 trials, with the use of dupilumab in patients with chronic rhinosinusitis with nasal polyps.^24^ Revision surgery is associated with more surgical risks than primary FESS because of major surgical complications, such as injury to the orbit or a cerebrospinal fluid leak.^10^ Of note, no major surgical complications following either primary FESS or revision surgery were reported in our study.

This study has limitations. It is retrospective in nature and relies on data recorded in medical notes. Not all patients had their peripheral blood eosinophil count, and total IgE and specific IgE responses tested; hence, there might be selection or sampling bias for the reported results. Information on patients having asthma or aspirin-exacerbated respiratory disease might have been missing, and some patients might therefore have been marked as having chronic rhinosinusitis with nasal polyps only or chronic rhinosinusitis with nasal polyps plus asthma. Data on medical polypectomy prescribed in the community by general practitioners were not available, and so there might be a higher percentage of patients who had medical polypectomy following FESS.

When chronic rhinosinusitis with nasal polyps plus asthma patients were given oral corticosteroids for asthma in the follow-up period, this was not counted as medical polypectomy, as the indication was for asthma, not chronic rhinosinusitis with nasal polyps. Some authors would count this as medical polypectomy, but in this study it was not as the primary indication for oral corticosteroids was not chronic rhinosinusitis with nasal polyps.

The study concentrated on patients who underwent FESS; therefore, some patients, who were candidates for the surgery but for unknown reasons did not undergo it, were missed from the overall analysis of the phenotype of patients with severe chronic rhinosinusitis with nasal polyps requiring FESS. Patients who underwent FESS in the private sector were also missed, and so there might be a higher number of patients with chronic rhinosinusitis with nasal polyps who underwent FESS and/or required revision surgery than is reported by us.

It was not possible precisely to assess patients having allergic rhinitis from clinical notes as symptoms and diagnosis were not mentioned accurately. Although information was gathered on the use of antihistamines as a surrogate, it is likely there is a proportion of patients who were taking over-the-counter antihistamines and the use of these was not recorded anywhere in the medical notes.

- Some patient factors are helpful in predicting severe recurrence of nasal polyps following functional endoscopic sinus surgery (FESS)
- Checking peripheral blood eosinophils and total immunoglobulin E is helpful in identifying patients with the highest likelihood of nasal polyp recurrence following FESS
- Computed tomography sinus scans help in planning FESS steps and assist in predicting severe nasal polyps recurrence following FESS
- Three distinct chronic rhinosinusitis with nasal polyps groups exist that might help predict severe nasal polyps recurrence following FESS
- This distinction is based on the presence or absence of asthma or aspirin-exacerbated respiratory disease
- Patients with a higher risk for revision surgery should require closer follow-up to maximise the most adequate therapy

The reported numbers of chronic rhinosinusitis with nasal polyps patients who had medical polypectomy or revision surgery following FESS is believed to be potentially lower than they actually were. There is no information on the patients who were not referred back to the ENT clinic despite having troublesome chronic rhinosinusitis with nasal polyps symptoms and/or who chose to see ENT doctors in the private sector. As part of this retrospective study, it was not possible to analyse the exact role of the extent of FESS and the need for medical polypectomy and/or revision surgery.

## Conclusion

Considering patients’ relevant past medical history, commonly available blood tests in addition to endoscopy and imaging can help clinicians to identify patients who have better or worse nasal polyp recurrence prognosis requiring medical polypectomy and/or revision surgery in future. Patients with a higher risk for revision surgery might require closer follow-up to maximise the most adequate therapy, including consideration for treatment with biologics such as dupilumab.

## Supplementary material

For supplementary material accompanying this paper visit https://doi.org/10.1017/S0022215123000853. click here to view supplementary material
