# Supplementary material for: Phenotypic associations of medical polypectomy and revision surgery following endoscopic sinus surgery: a retrospective study of a single-centre experience in Scotland
Source: J Laryngol Otol. 2023 May 22;137(11):1277–84. doi: 10.1017/S0022215123000853 (PMC10627783; doi:10.1017/S0022215123000853)
Supplement: Supplementary file 1 [file S0022215123000853sup001.docx]

<JLO 23-0050; supplementary material>

**Table 1.** Extent of performed FESS

| Part of FESS performed | MMA | Ethmoidectomy | | Sphenoidotomy | Frontal recess clearance |
| --- | --- | --- | --- | --- | --- |
|  |  | Anterior | Posterior |  |  |
| Yes | 195 (98.0%) | 155 (77.8%) | 133 (66.8%) | 70 (35.2%) | 99 (49.7%) |
| No | 4 (2.0%) | 44 (22.1%) | 66 (33.2%) | 129 (64.8%) | 100 (50.3%) |

Data available for 199 patients. FESS = functional endoscopic sinus surgery; MMA = middle meatal antrostomy

**Table 2.** Adjusted odds ratios for medical polypectomy, revision FESS and combined polypectomy

| Parameter | Medical polypectomy | Revision surgery | Combined polypectomy |
| --- | --- | --- | --- |
| Younger than 55 years of age | 2.7 (1.3,5.6) ** | 8.9 (2.0,39.5) ** | 3.0 (1.5,6.1) ** |
| History of at least one previous FESS | 2.8 (1.4,5.8) ** | 5.8 (1.9,17.9) ** | 3.3 (1.6,6.6) ** |
| Average  PBE ≥300 per µl | 3.7 (1.4,9.5) ** | 3.1 (0.8,12.5) | 3.7 (1.5,9.2) ** |
| LMS >17 | 2.4 (1.1,5.6) * | 9.1 (2.3,36.4) ** | 2.6 (1.2,5.9) * |
| CRSwNP + AERD compared to CRSwNP-only | 11.5 (3.8,35.1) *** | 13.8 (3.6,52.7) *** | 13.3 (4.6,38.9) *** |

Data represent adjusted odds ratios (95 per cent confidence intervals). **p* < 0.05 ***p* < 0.01 ***p<0.001. CRSwNP = chronic rhinosinusitis with nasal polyps; AERD = aspirin-exacerbated respiratory disease; FESS = endoscopic sinus surgery; LMS – Lund–Mackay score; PBE = peripheral blood eosinophils

**Table 3.** Comparison between CRSwNP-only and CRSwNP + asthma (including AERD) patients

| Parameter | CRSwNP-only | CRSwNP + asthma (including AERD) | *p-*value |
| --- | --- | --- | --- |
| Number of patients (% of total) | 108 (48.9%) | 113 (51.1%) |  |
| Male : female (ratio) | 85 : 23 (3.7 : 1) | 79 : 34 (2.3 : 1) | 0.166 |
| Female (% of all patients) | 23 (10.4%) | 34 (15.4%) |  |
| Female (% of all female patients) | 23 (40.4%) | 34 (59.6%) |  |
| Male (% of all patients) | 85 (38.4%) | 79 (35.7%) |  |
| Male (% of all male patients) | 85 (51.8%) | 79 (48.2%) |  |
| Age ± SD | 52.7 ± 14.0 | 53.1 ± 13.8 | 0.311 |
| Average PBE ± SD, cells in µL | 314 ± 151 (n = 80) | 419 ± 201 (n = 96) | <0.001 |
| Total IgE | 56 (n = 36) | 120 (n = 53) | 0.006 |
| NPS (IQR) | 6 (1) (n = 95) | 7 (2) (n = 103) | 0.012 |
| LMS ± SD | 12.2 ± 5.1 (n = 94) | 14.6 ± 5.3 (n = 84) | <0.001 |
| Have not had previous FESS (% of each group total) | 80 (74.1%) | 52 (46%) | <0.001 |
| Have had previous FESS (% of each group total) | 28 (25.9%) | 61 (54%) | <0.001 |
| Number of previous FESS per patient | 0.53 | 1.37 | 0.004 |
| Medical polypectomy | 14 (13.7%) | 30 (29.4%) | 0.010 |
| Revision surgery | 5 (4.9%) | 14 (13.1%) | 0.053 |
| Combined polypectomy | 16 (15.5%) | 35 (32.7%) | 0.004 |

Data are shown as arithmetic means aside from immunoglobulin E as geometric mean and nasal polyp score as median. AERD = aspirin-exacerbated respiratory disease; CRSwNP = chronic rhinosinusitis with nasal polyps; FESS = functional endoscopic sinus surgery; IQR = interquartile range; LMS = Lund–Mackay score; NPS = nasal polyp score; PBE = peripheral blood eosinophils; SD = standard deviation

**Table 4.** Comparison between CRSwNP patients with asthma (non-AERD) versus AERD

| Parameter | CRSwNP + asthma | CRSwNP + AERD | *p-*value |
| --- | --- | --- | --- |
| Number of patients (% of total) | 86 (38.9%) | 27 (12.2%) |  |
| % of patients of asthma/AERD combined group | 76.1% | 23.9% |  |
| Male : female (ratio) | 62 : 24 (2.6 : 1) | 17 : 10 (1.7 : 1) | 0.471 |
| Female (% of all patients) | 24 (10.1%) | 10 (4.5%) |  |
| Female (% of all female patients) | 24 (42.1%) | 10 (17.5%) |  |
| Male (% of all patients) | 62 (28.1%) | 17 (7.7%) |  |
| Male (% of all male patients) | 62 (37.8%) | 17 (10.4%) |  |
| Age ± SD | 54.2 ± 13.9 | 49.7 ± 13.1 | 0.0137 |
| Average PBE ± SD, cells in µl | 392 ± 191 (n = 73) | 514 ± 233 (n = 23) | 0.013 |
| Total IgE | 110 (n = 33) | 138 (n = 20) | 0.516 |
| NPS (IQR) | 7 (2) (n = 81) | 7 (2) (n = 22) | 0.6 |
| LMS ± SD | 14.1 ± 5.3 (n = 71) | 17.7 ± 4.5 (n = 13) | 0.022 |
| Have not had previous FESS (% of each group total) | 50 (58.1%) | 2 (7.4%) | <0.001 |
| Have had previous FESS (% of each group total) | 36 (41.9%) | 25 (92.6%) | <0.001 |
| Number of previous FESS per patient | 0.83 | 3.11 | <0.001 |
| Medical polypectomy | 17 (20.7%) | 13 (65%) | <0.001 |
| Revision surgery | 5 (6%) | 9 (37.5%) | <0.001 |
| Combined polypectomy | 18 (21.7%) | 17 (70.8%) | <0.001 |

Data are shown as arithmetic means aside from immunoglobulin E as geometric mean and nasal polyp score as median. AERD = aspirin-exacerbated respiratory disease; CRSwNP = chronic rhinosinusitis with nasal polyps; FESS = functional endoscopic sinus surgery; IQR = interquartile range; LMS = Lund–Mackay score; NPS = nasal polyp score; PBE = peripheral blood eosinophils; SD = standard deviation
